# Hormone replacement therapy in surgical menopause after gynecological malignancies

**DOI:** 10.17305/bb.2024.11220

**Published:** 2024-11-15

**Authors:** Dragana Tomić Naglić, Aljoša Mandić, Milica Zirojević, Nikolina Vuković, Sladjana Pejaković, Mia Manojlovic, Ivana Bajkin, Tijana Ičin, Stefan Janičić, Edita Stokić

**Affiliations:** 1 University of Novi Sad, Faculty of Medicine in Novi Sad, Novi Sad, Serbia; 2 Clinic for Endocrinology, Diabetes and Metabolic Disorders, Clinical Center of Vojvodina, Novi Sad, Serbia; 3 Institute of Oncology of Vojvodina, Sremska Kamenica, Serbia

**Keywords:** Hormone replacement therapy, HRT, surgical menopause, gynecological malignancies, quality of life, cardiovascular risk, bone health

## Abstract

This review examines hormone replacement therapy (HRT) in cases of surgical menopause following gynecological malignancies. It aims to capture current knowledge, summarize recent findings, and provide recommendations for clinical settings. Unlike natural menopause, surgical menopause occurs abruptly, without an adjustment period, and is associated with a notably higher risk of fractures, arthritis, cognitive decline, dementia, Parkinson’s disease, and various metabolic disorders affecting glucose and lipid levels—all of which contribute to an increased risk of major cardiovascular events. In 2017, The North American Menopause Society recommended that, barring contraindications, HRT should be initiated in women who enter surgical menopause before age 45. If these women do not experience vasomotor symptoms or other issues, HRT should be maintained consistently at least until age 52. This guideline reflects contemporary knowledge and is the result of a multidisciplinary consensus, based on a review of existing literature and several randomized clinical trials focusing on women who have survived gynecological cancers and whose quality of life is significantly impacted by surgical or early menopause. Estrogen supplementation is particularly beneficial, as it is linked to marked improvements in quality of life, including, delayed onset of chronic cardiovascular issues, reduced fracture risk, enhanced cognitive function, reduced inflammation, and improved self-esteem, as well as better social and work performance. Clinical implementation of HRT, however, requires a highly individualized approach. This approach must consider the type and stage of malignancy, histopathological characteristics, risk factors for recurrence (such as diet, concurrent medications, medical history, and genetic predispositions), and a thorough assessment of the potential benefits and risks of HRT, as well as the patient’s personal wishes and expectations.

## Introduction

This review article was developed in collaboration with gynecologic oncologists and endocrinologists. The idea for this work was inspired by recent innovative findings that call for a paradigm shift in managing patients with gynecological malignancies. Advances in the diagnosis and treatment of cancer have significantly improved survival rates, emphasizing the need to focus on the quality of life for survivors post-treatment.

For this literature review, only studies conducted on human subjects were considered. Given the sensitive nature of the topic—survivorship following gynecological malignancies—the insights and data were primarily drawn from patient registries, as randomized clinical trials in this area are scarce. To further validate the findings, we also incorporated guidelines and recommendations from leading organizations in the field. The primary database used for this search was PubMed, with keywords including, “gynecological malignancy,” “hormone replacement therapy (HRT) after gynecological malignancy,” “hormone replacement therapy HRT ovarian cancer,” “BRCA1 carrier,” “BRCA2 carrier,” “estrogen,” and “endometrial cancer.” Additional searches were conducted on Google Scholar and Web of Science.

The approach to addressing gynecological malignancies is centered on five key principles:

1. *Prevention of disease:* Optimizing nutrition, smoking cessation, and limiting consumption of coffee and tea [1]. HPV vaccination: The nonavalent HPV vaccine can prevent 90% of cervical cancers, 61% of vaginal cancers, 23% of vulvar cancers, as well as other cancers, such as laryngeal, oropharyngeal, penile, anal, and oral cavity cancers [2, 3]. Educating patients on sexually transmitted diseases and the importance of stabilizing the vaginal microbiome [2, 3]. Promoting maintenance of healthy body weight and treating all components of cardiometabolic syndrome in overweight or obese individuals [4, 5].
2. *Early screening of disease:* Encouraging regular gynecological examinations, with increased surveillance for individuals who: had an earlier sexual debut; have multiple sexual partners; suffer from autoimmune diseases; and are on immunosuppressive therapy or are immunodeficient [6].
3. Diagnosis and staging of the disease.
4. Surgical and adjuvant treatment.
5. Quality of life and psychosocial adjustment for cancer survivors [1–7].

The GLOBOCAN 2020 project identifies breast, cervical, ovarian, and uterine cancers as some of the ten most prevalent cancers affecting women globally. In 2020, gynecological cancers were estimated to be the fourth most common cancer diagnosis worldwide, with 604,000 new cases reported. Additionally, these cancers accounted for 342,000 deaths, making them the fourth leading cause of cancer-related mortality in women [8]. Recent epidemiological studies indicate that 56% of gynecological cancer patients are between the ages of 30 and 49, with an average age of 44.84 years. Approximately 31% of these cancers are diagnosed in stages I and II, and 25% are confirmed as well-differentiated based on histological findings [9].

Among the major gynecological malignancies—excluding breast cancer—cervical cancer remains a significant concern. According to a large-scale epidemiological study by Yi et al., the incidence, mortality, and disability-adjusted life years (DALYs) associated with cervical cancer steadily increased worldwide between 1990 and 2019. However, global trends show that both incidence and mortality rates for cervical cancer have declined over the past 30 years. Ovarian cancer, in contrast, has consistently had a lower incidence globally. Meanwhile, the incidence of uterine cancer has risen, though its mortality rate has declined during the same period. The study also highlights regional differences: breast cancer remains the most frequently diagnosed cancer in women worldwide, while cervical cancer is more common in areas with a low socio-demographic index (SDI). Conversely, ovarian and uterine cancers are more prevalent in regions with a high SDI [10].

The approach of providing comprehensive care to patients with gynecological cancer, spanning from diagnosis to end-of-life care, is not a novel concept. One of the earliest publications addressing this topic appeared in 2006, titled “From Cancer Patient to Cancer Survivor: Lost in Transition” [11]. Research conducted from 2006 to 2020 has shed light on the unique needs of gynecological cancer survivors and their caregivers. These individuals require a distinct approach from healthcare professionals—one that emphasizes detailed guidance on monitoring for potential cancer recurrences and support in addressing various health and psychosocial challenges. Such challenges include anxiety, depression, sleep disturbances, cognitive impairments, interpersonal relationship changes, and post-traumatic stress syndrome [12].

Among the most clinically significant conditions affecting younger patients is artificial menopause, often managed collaboratively by gynecologists and endocrinologists. This condition profoundly diminishes quality of life, exacerbated by the absence of a natural menopausal transition. Given these additional psychosocial and physical difficulties, all women treated for gynecological malignancies should receive professional support to navigate these challenges. This support should adopt a holistic perspective, grounded in modern guidelines, and evidence-based medicine [13].

### Artificial menopause

Cardiovascular risk in women rises significantly during menopause, reaching levels comparable to those in men. This risk is influenced by the age at menopause onset, with younger age at onset being associated with higher risks. Interestingly, this relationship appears to be bidirectional: women with pre-existing cardiovascular conditions are more likely to experience early menopause or premature ovarian failure (POF) [14].

Cardiovascular risk is particularly elevated in cases of post-surgical menopause (e.g., hysterectomy with bilateral ovariectomy) compared to natural menopause. In many cases, the treatment of gynecological malignancies in women of reproductive age results in surgical menopause, which is marked by a sudden and dramatic decline in estrogen levels. Unlike physiological menopause—typically occurring between the ages of 46 and 58, with a preceding adaptive period of menopausal transition—surgical menopause lacks this adjustment phase. As a result, women who undergo surgical menopause experience more pronounced vasomotor symptoms.

Menopause occurring before the age of 45 is categorized as premature, while menopause before 40 is classified as premature ovarian insufficiency. These conditions carry a significantly elevated risk, not only for chronic cardiovascular diseases but also for fractures, arthritis, cognitive decline, dementia, Parkinson’s disease, and metabolic disorders affecting glucose and lipid metabolism. These metabolic disruptions further increase the risk of major cardiovascular events [15].

Additionally, adjuvant treatments, such as chemotherapy and radiotherapy can worsen the quality of life for women treated for gynecological malignancies. Notably, more than half of gynecological oncology patients are premenopausal at the time of diagnosis, and at least one-third are treated and cured in stage I or II of the disease with a favorable histopathological cancer type. This raises important questions regarding the quality and longevity of life for young oncology patients [16].

HRT is based on two principles:

- Relieving the patient’s symptoms
- Preventing the effect on comorbidities [15].

*Benefits of HRT [13]:*

- The effect on vasomotor inconveniences, which in women in the menopausal transition last about seven years, but in 20% of cases, the length is up to 15 years.
- Sexual dysfunction (other than estrogen products with or without progesterone, possible inclusion of androgens (testosterone) and DHES).
- Effect on genitourinary syndrome (vaginal dryness, dyspareunia, bladder wall atrophy, and incontinence).
- Emotional state.
- Musculoskeletal effects (complaints are often declared as fibromyalgia symptoms).
- Osteoporosis prevention.
- Cardiovascular diseases prevention, if treated on time.
- Impact on cognition.

Studies so far indicate that cognitive disorders occur in 70% of postmenopausal women, and cognitive decline occurs as soon and rapidly after entering menopause. The most common disorder is difficulty learning new words or recalling previously learned words and numbers [13].

Notably, “The Mayo Clinic Cohort Study of Oophorectomy and Aging” highlights a significant relationship between oophorectomy and cognitive disorders. According to the study, unilateral ovariectomy performed before the age of 41 doubles the risk of cognitive impairment. For patients younger than 34, this risk can quadruple. However, the use of estrogen products following oophorectomy can reduce these risks. If there are no contraindications, estrogen therapy is recommended at least until the age of 50 [17].

### The role of estrogens in the prevention of menopausal comorbidities and the promotion of well-being

#### Estradiol and hot flashes

Estradiol plays a critical role in thermoregulation in women during their fertile years by reducing the activity of kisspeptin, neurokinin B, dynorphin (KNDy) neurons via its estrogen receptor alpha (ERα) receptors. During menopause, the decline in estrogen levels results in increased KNDy activity and heightened expression of neurokinin 3 receptors (NK3Rs) in the medial preoptic nucleus, which disrupts thermoregulation. Postmenopausal KNDy neurons also become hypertrophic. Adequate estradiol replacement therapy in postmenopausal women can restore physiological thermoregulation by suppressing excessive KNDy activity. However, because HRT is contraindicated in cases of advanced gynecological malignancies, clinical trials are investigating the use of NK3 receptor blockers as a potential treatment to alleviate hot flashes [18, 19].

#### Neuroprotection

Globally, 11%–18% of people suffer from mental disorders, with the proportion being significantly higher among women [20]. Estrogen plays a key role in regulating neurotransmitters, such as glutamate, dopamine, and serotonin, thereby influencing cognitive abilities, mood, working memory, and the brain’s reward system [21]. Additionally, estrogen contributes to neuroprotection through its role in synthesizing neurotrophins and its anti-inflammatory properties, which help defend the nervous system against stress [21].

Estrogen receptors (ER) are distributed throughout the brain, with ERβ predominantly expressed in the frontal cortex, sensorimotor cortex, thalamus, and cerebellum. In contrast, ERα has a dominant role in brain regions associated with learning and memory, such as the hippocampus. ERα is primarily localized in the nucleus, while ERβ resides in the cytoplasm [22].

On a global scale, women over 65 are nearly twice as likely as men of the same age to develop Alzheimer’s disease. Risk factors for this form of dementia include genetics, diet, age, education, social factors, gender, and chronic illnesses. The higher prevalence of Alzheimer’s among women can be partially attributed to fluctuations in estrogen levels between the childbearing years and menopause [23].

According to the data available so far, the following neuroprotective mechanisms of estrogen can affect the appearance of this form of dementia:

- The rapid decline in estrogen during menopause accelerates demyelination; HRT helps regulate the production of new myelin [24].
- Animal models have shown that estrogen replacement in ovariectomized individuals inhibits the formation of human tau (tau) protein [25].
- Estrogen reduces neurotoxicity by increasing the levels of thioredoxin, which reduces oxidative stress-induced cell death [26].
- Estradiol also significantly impacts the mitochondrial level, stimulating glycolysis, and gene transcription [27].

#### The impact of estradiol on weight gain

Studies conducted so far have established that an estradiol deficit contributes to lean mass reduction. However, this issue can be mitigated through the use of transdermal hormone replacement therapies after menopause [28]. The underlying mechanism of this phenomenon is linked to the decrease in GnRH levels during HRT, which prevents a reduction in resting energy expenditure (REE) [29, 30]. In contrast, during menopause, the decline in estradiol levels and the corresponding increase in GnRH stimulate orexigenic neuropeptide Y (NPY) neurons. Interestingly, while GnRH neurons themselves do not express ERα, feedback related to estradiol levels is mediated through other neurons connected to GnRH neurons.

Recent research has focused on ERα-expressing kisspeptin neurons located in the arcuate nucleus (ARH) of the hypothalamus, which are now recognized as key regulators of GnRH pulse release. Notably, the ARH also plays a critical role in energy homeostasis by maintaining elevated REE levels. In cases of estrogen deficiency and inadequate ARH stimulation, REE levels decrease, leading to increased appetite [31].

**Figure 1.**
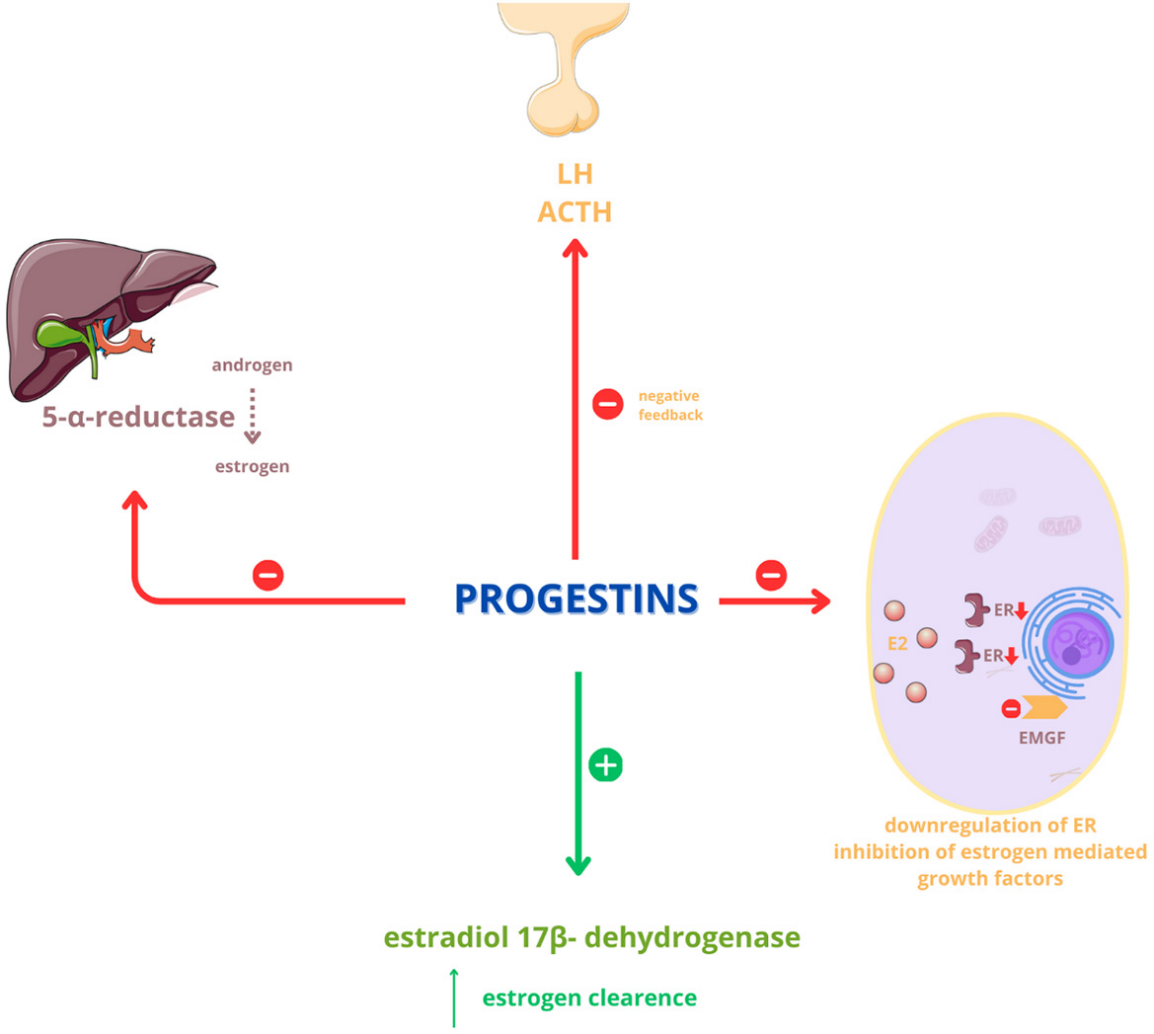
Anti-estrogenic effects of progestins.

#### Estrogen and bone metabolism

HRT with estrogen is approved for managing moderate to severe menopausal vasomotor symptoms and for preventing osteoporosis [32]. Estrogen plays a crucial role in bone development and density. Its deficiency during childhood delays skeletal growth, while in adulthood, it contributes to the development of osteoporosis. Estrogen supports bone metabolism by promoting the proliferation of plate chondrocytes, increasing bone mass accumulation, and maintaining bone density. It acts directly on bone cells and indirectly influences calcium balance. Estrogen deficiency reduces calcium absorption in the intestines and causes a “renal calcium leak,” which can trigger symptoms of secondary hyperparathyroidism [33].

#### Estrogen and inflammation

The study conducted by Averyanova et al. highlights an increase in inflammatory cytokines, such as TNF-α, IL-β, IL-6, and IL-8, along with significant changes in the immune system, including alterations in the proportions of CD4+ and CD8+ T lymphocytes. These findings suggest a molecular link between estrogen deficiency and elevated levels of inflammation [34]. Conversely, recent research in the field of immunosenescence has confirmed that estrogen supplementation in menopausal women reduces these pro-inflammatory cytokine levels [35].

### Recommendations

In 2017, The North American Menopause Society recommended that, in the absence of contraindications, HRT should be initiated for women who experience surgical menopause before the age of 45. This recommendation applies even if these women do not exhibit vasomotor symptoms or other complaints, with the therapy advised to continue at least until they reach 52 years of age—the average age at which women typically enter natural menopause [36]. Additionally, the Society addressed strategies for HRT usage in patients with gynecological malignancies [37].

Similarly, the British Menopause Society issued a consensus in 2021 with recommendations on HRT in menopause (Table 1, [13], [16], [36], [38–52]). This document provides a clear outline of the risks associated with HRT, particularly in relation to cancer development, while also weighing the benefits and drawbacks of HRT for women with a history of gynecological malignancy [13]. A revised version of this consensus is anticipated in 2025.

**Table 1:** Overview of HRT risks, usage, and guidelines in gynecologic oncology

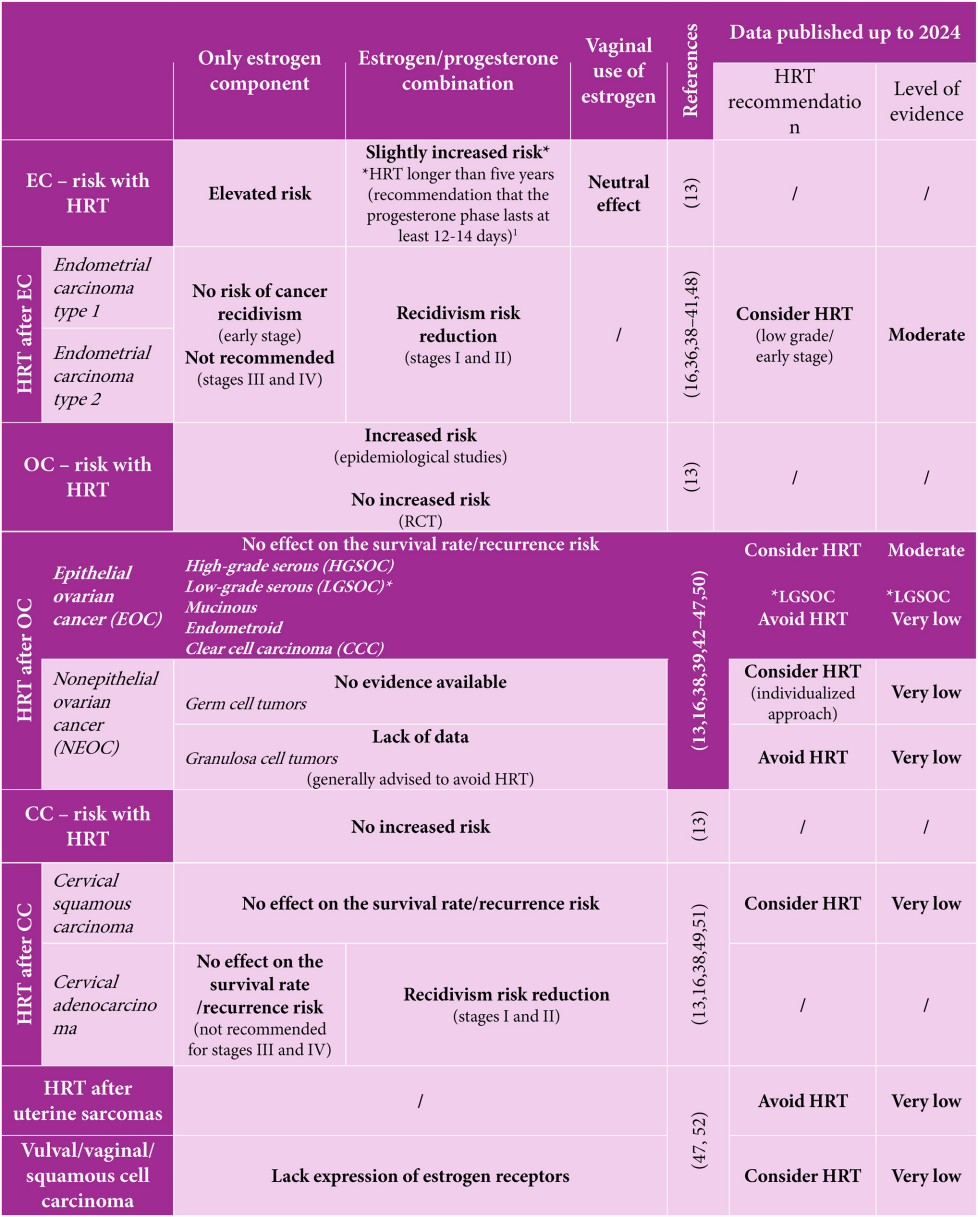

HRT: Hormone replacement therapy; RCT: Randomized controlled trial.

The significance of incorporating progesterone preparations into HRT following endometrial cancer lies in progesterone’s anti-estrogenic effects, which are achieved through several mechanisms (Figure 1).

- It inhibits the process of converting androgens into estrogens in the liver by inhibiting the enzyme 5-α-reductase;
- It increases the clearance of estradiol via 17-β-hydrogenase;
- It inhibits estrogen-mediated growth factors;
- Negative feedback at the pituitary level inhibits the secretion of LH and ACTH [53–54].

As Eeles et al. [55] noted in their 2015 publication, data from numerous studies monitoring the use of HRT—whether as an estrogen-only component or combined estrogen/progesterone therapy—for at least five years prior to the diagnosis of epithelial ovarian cancer serves as a strong prognostic criterion for patient survival. In contrast to earlier views suggesting that HRT increases the risk of ovarian cancer, Brieger et al. [56] studied a sample of 6419 individuals with advanced malignant ovarian disease and demonstrated that menopausal HRT use exceeding five years positively impacted survival, with an average survival time of 5.75 years compared to 4.6 years in non-HRT users. Despite these findings, current guidelines recommend HRT only after curing stages I and II of the disease. This data underscores the need to promote HRT among healthy menopausal women under the age of 60.

Prospective randomized clinical trials have also shown that HRT use following surgical treatment for ovarian cancer is associated with favorable prognostic outcomes. A 2020 meta-analysis reinforced these findings, confirming HRT’s positive impact on survival rates [57].

More recently, in 2022, Ji et al. analyzed data from a cohort of 1784 women who underwent surgical treatment for ovarian cancer, with or without additional neoadjuvant therapy. The study excluded individuals over 60 years old, those with pre-diagnosis venous thromboembolism, and those with concurrent malignancies in other locations. The average age of patients receiving HRT (*n* ═ 263) and the control group without HRT (*n* ═ 1521) was 41 years. Over a follow-up period of 5.6 ± 2.9 years, the results revealed longer survival rates for HRT users post-treatment compared to non-users (85.3% vs 76.6%; *P* ═ 0.016). Additionally, the analysis showed that longer durations of HRT use were associated with even greater survival benefits [58].

In the interim, observational studies, randomized clinical trials, and meta-analyses have examined the relationship between HRT and histopathological characteristics of ovarian carcinoma. These studies, with follow-up periods ranging from 42 months to 19 years, have produced further recommendations detailed below.

Findings published in 2024, though not yet incorporated into current guidelines, draw upon data from gynecologic oncology and may assist healthcare providers in determining whether to incorporate HRT into patient care plans. Table 1, adapted from Hickey et al., provides a summary of these results.

### Contraindications

#### HRT after endometrial cancer

There is insufficient data on the safety of hormone therapy for advanced stages (III–IV) of endometrial cancer or uterine sarcoma. Consequently, hormone therapy is generally not recommended [37, 59]. While meta-analyses based on retrospective studies and one randomized controlled trial (RCT) did not indicate adverse effects, recurrence, or reduced survival [48, 60, 61], systemic hormone therapy remains inadvisable for cases of high-grade, advanced-stage endometrial cancer, endometrial stromal sarcoma, or leiomyosarcoma due to the limited evidence available regarding its safety [37].

### HRT after ovarian cancer

Hormone therapy is generally not recommended for patients with hormone-sensitive cancers, such as low-grade serous carcinoma, endometrioid carcinoma, or granulocytic carcinoma [37, 62]. Borderline tumors, which are more common in younger women, are associated with high survival rates. While research on this topic is limited, hormone therapy may be appropriate for women who have achieved complete surgical remission, particularly in cases of early menopause where its benefits can outweigh potential risks [63]. However, a rare form of ovarian cancer, malignant mixed mesodermal tumor, is a contraindication for HRT [64]. Notably, the majority of studies suggest that HRT does not increase cancer risk and may even reduce it. Accordingly, guidelines from the DGGG, OEGGG, and SGGG recommend that HRT can be considered to address significant declines in quality of life, provided all relevant factors are thoroughly evaluated [65].

### HRT after cervical cancer

Current studies indicate that HRT is used in fewer than 50% of cases following cervical carcinoma when treatment results in artificial menopause. Even when HRT is initiated, its duration is often insufficient to significantly improve patients’ quality of life or delay comorbidities [66]. Notably, HRT is considered safe for patients with a history of cervical cancer [37, 62]. ER positivity does not influence cervical cancer prognosis.

Over 80% of cervical cancer cases are classified as squamous cell carcinoma, which is known to be estrogen-independent. One RCT involving 120 patients under the age of 45 with stage I and II cancer was identified. Over an observation period exceeding five years, oral HRT was combined with various formulations. No significant differences in mortality (RR 0.62, 95% CI: 0.33–1.15) or recurrence (RR 0.57, 95% CI: 0.31–1.15) were observed [49].

In contrast, 10%–20% of cervical cancer cases are adenocarcinomas, which show a significantly increased risk associated with estrogen monotherapy (OR ═ 2.7; 95% CI: 1.1–7.9). However, with combined HRT, this risk is reduced or negligible (RR 1.1; 95% CI: 0.26–5.0) [67]. Given the biological similarities to endometrial cancer, a combined estrogen–progestogen regimen is recommended.

A systematic review by Vargiu et al. concluded that HRT is not contraindicated in cervical cancer survivors. For patients with an intact uterus, the addition of progestogen is advised to protect the endometrium, as endometrial tissue may remain viable despite prior pelvic radiation or chemotherapy [67].

### HRT after sarcoma

Gynecological sarcomas are highly aggressive tumors often diagnosed at a late stage, with a 5-year survival rate ranging from 31% to 64% [68]. Uterine sarcomas include leiomyosarcomas (uLMS), rhabdomyosarcomas (RMS), adenosarcomas, low-grade endometrial stromal sarcomas (LGESS), high-grade endometrial stromal sarcomas (HGESS), undifferentiated uterine sarcomas (UUS), and perivascular epithelioid cell tumors (PEComas). Endometrial stromal sarcomas overexpress estrogen and progesterone receptors; therefore, estrogenic HRT and tamoxifen can negatively influence disease progression. As a result, avoiding HRT in these cases is generally recommended, although the evidence is limited [38].

Leiomyosarcomas also frequently express estrogen and progesterone receptors, but the data on their hormone sensitivity remain inconclusive. For instance, in the study by Kapp et al., bilateral ovariectomy during hysterectomy did not impact five-year survival, suggesting that leiomyosarcomas may not be hormone-sensitive. This leaves the theoretical possibility for HRT use, though many consider it too risky due to a lack of direct evidence supporting its safety. Conversely, HRT may be used in cases of carcinosarcoma and adenosarcoma [69, 70].

### Barrier

One of the most prominent controversies surrounding HRT stems from the findings of the Women’s Health Initiative Memory Study (WHIMS). While earlier studies suggested that HRT reduces the risk of Alzheimer’s disease by 30%, WHIMS reported contradictory results, indicating that the risk of developing neurodegenerative diseases doubles [71]. However, because WHIMS focused exclusively on women over the age of 65, concerns arose about the applicability of its findings to the broader population of postmenopausal women.

Subsequent clinical trials, including, MIRAGE, Study of Women’s Health Across the Nation (SWAN), and REMEMBER, addressed this issue by stratifying participants based on their age and the timing of HRT initiation relative to menopause onset. These studies consistently concluded that starting HRT as early as possible after menopause significantly improves cognitive function [72].

The disparity in WHIMS findings is largely explained by its study population, which consisted of women who had experienced prolonged estrogen deprivation. ERs, located in regions, such as the cerebral cortex, hypothalamus, and hippocampus, play a key role in synaptogenesis and neuronal plasticity. In menopause, this deprivation reduces the brain’s ability to form new synapses, contributing to cognitive decline [72–74].

Further evidence from the SWAN highlights the benefits of initiating HRT even before menopause is clinically diagnosed. SWAN suggests that starting therapy during the perimenopausal period—at the onset of early symptoms—has a significantly greater positive effect on cognition than delaying treatment until after menopause [75].

In 2023, a meta-analysis was published addressing the long-standing concern that HRT increases the risk of ovarian malignancy, particularly since most epithelial ovarian cancers are ER-positive. This analysis summarized the results of 11 studies. Notably, these studies did not stratify patients by disease stage, tumor resectability, adjuvant therapy, histological type, or degree of differentiation of the ovarian cancer. Despite this lack of stratification, the meta-analysis demonstrated with high statistical significance (*P* < 0.00001) that HRT provides a survival advantage, irrespective of disease stage or cancer histology. The findings showed improved overall survival and length of survival, along with a positive impact on menopause symptom suppression and quality of life [76]. However, despite these results, the authors of the publication argue that HRT in patients with ovarian malignancy should only be considered in younger patients who are in good general health and have a better prognosis. This perspective aligns with current guidelines, which restrict the use of HRT to patients with stage I or II ovarian cancer.

Current guidelines recommend bilateral salpingo–oophorectomy to reduce the risk of malignancy in BRCA1 mutation carriers after completing reproduction or by the age of 35–40. For BRCA2 mutation carriers, the procedure is advised between ages 40 and 55. This results in at least two-thirds of patients experiencing POF or early menopause, which is associated with a reduction in average life expectancy by 3.1 years (95% CI, −5.1 to −1.1) and an increased risk of related symptoms and comorbidities [58].

Loizzi et al. recommend that patients with BRCA1 or BRCA2 mutations begin short-term HRT immediately after preventive bilateral salpingo–oophorectomy and continue it until the age of 51. Beyond age 51, HRT may be indicated for women experiencing persistent symptoms after discontinuing therapy. However, Loizzi et al. also stress the need for additional clinical studies to better understand the benefits of HRT in this population [77].

Healthcare professionals approach this type of therapy with caution. A survey of doctors, oncologists, and gynecologists involved in the prevention and treatment of malignancies in patients with BRCA mutations revealed that, in most cases, HRT is initiated at the patient’s request rather than being recommended by the doctor. The survey also highlighted differences in how various specialists view the safety of HRT in specific contexts. For instance, gynecologists and oncologists generally consider HRT safe after cervical cancer. However, only 50% of respondents support its use following ovarian cancer, despite the lack of clear contraindications. Furthermore, two-thirds of the participants do not recommend HRT after endometrial cancer, even in its early stages. Despite these reservations, some healthcare professionals recognize the potential benefits of HRT in improving quality of life and preventing chronic complications [39].

One of the most pressing controversies surrounding HRT is the inadequate awareness of its importance in disease prevention and symptom management within the general population. This issue was explored by Grandi et al., who concluded that awareness of HRT’s benefits is notably lacking among women with BRCA1 or BRCA2 mutations. Simultaneously, concerns about the risks associated with HRT, particularly its potential oncogenicity, are often exaggerated and largely unfounded [78]. These apprehensions are shared by some healthcare professionals, leading to patients receiving contradictory advice. Therefore, it is crucial to intensify efforts to educate not only the general female population but also women with oncogenic mutations and healthcare providers. This education should focus on dispelling existing prejudices, myths, and taboos surrounding HRT.

## Conclusion

The guideline is based on contemporary knowledge and the consensus of a multidisciplinary team, derived from empirical data on patients who underwent HRT after surviving gynecologic malignancies.

Its clinical implementation necessitates a highly individualized approach, centered on confirming the type and extent of malignancy, histopathological characteristics, risk factors for recurrence (including nutritional factors, concurrent medications, medical history, and genetic predisposition), and conducting a thorough evaluation of the potential benefits, risks of HRT, and the patient’s personal preferences and expectations.

The limited number of randomized clinical studies in this field highlights the pressing need for further research to establish a more robust foundation for future recommendations. Nonetheless, therapy must remain patient-centered, prioritizing improved quality of life and anticipated lifespan post-treatment and recovery. Current challenges include gaps in knowledge and ongoing debates regarding HRT use in individuals treated for breast cancer, particularly those with receptor-negative forms of the disease. However, promising preliminary research in this area is emerging.
